# Corrosion behavior of 904L austenitic stainless steel in hydrofluoric acid

**DOI:** 10.1039/c7ra12453h

**Published:** 2018-01-12

**Authors:** Guobo Zou, Wei Shi, Song Xiang, Xuanming Ji, Guoqiang Ma, R. G. Ballinger

**Affiliations:** College of Materials and Metallurgy, Guizhou University Guiyang 550025 China sxiang@gzu.edu.cn; H. H. Uhlig Corrosion Laboratory, Massachusetts Institute of Technology Cambridge MA 02139 USA

## Abstract

Corrosion behaviors of 904L austenitic stainless steel in HF and HCl were studied and compared using electrochemical method, microscopic analysis, and phase analysis. An insoluble layer is deposited on 904L in HF due to a preferential reaction between [F^−^] and the [Ni] from the alloy. This insoluble deposited layer not only helps isolate the aggressive ions from the base metal, but also inhibits the passivation of 904L in HF, the mechanism for which was entirely different from that in HCl.

## Abstract 2

An insoluble layer is deposited on 904L in HF due to a preferential reaction between [F^−^] and the [Ni] from the alloy. This insoluble deposit layer helps isolate the aggressive ions from the base metal, while inhibits the passivation of 904L in HF.

## Graphical abstract

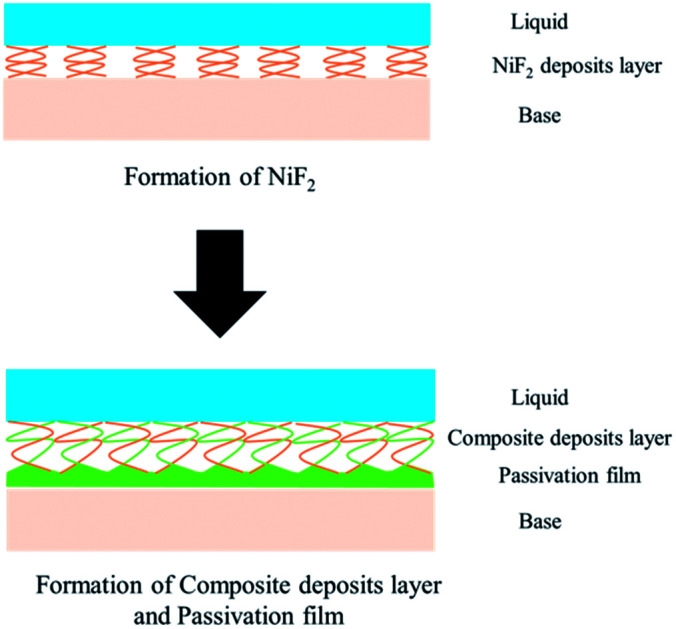

## 1. Introduction

Hydrofluoric acid (HF) is an extremely corrosive acid; thus, polymer materials (such as polytetrafluoroethylene) are widely used in industries using HF, and less attention has been directed towards the corrosion of metallic materials in HF. However, in some industries, the contact between metal and HF is inevitable. For instance, in the mineral processing industry, the powdered natural ore, containing calcium fluoride and other impurities, is dissolved in sulfuric acid, which leads to a HF–H_2_SO_4_ mixed acid system. Stainless steel could be passive in concentrated H_2_SO_4_; however, the effect of F^−^ on passivation was not clear.

There are few studies on stainless steel corrosion in HF. Mason^1^ found that certain amount of HF forms a stable iron fluoride on the metal surface, which effectively inhibits stainless steel corrosion by HNO_3_. Li^2^ investigated the effect of fluoride ion on the passivation properties of 316L stainless steel in acidic medium, and found that a fluorine-rich passivation film is formed on the stainless steel surface. By contrast, Stypula^3^ found that fluoride ions are adsorbed at the grain boundary edge in stainless steel, causing intergranular corrosion, thus accelerating local corrosion.

904L is a new type of high-chromium nickel–molybdenum super austenitic stainless steel with excellent corrosion resistance in acid environment.^4–6^ Pawel and Rebak *et al.* found that nickel-based alloys exhibited excellent corrosion resistance in HF.^7,8^ Although the content of nickel in 904L is 24 wt%, whether nickel can be the key factor for affecting the corrosion resistance of 904L in HF environment remains unclear. In this study, HCl environment was used as a reference system for 904L stainless steel in HF environment. The effect and mechanism of fluoride ions on the passivation process of 904L stainless steel were also investigated.

## 2. Experimental methods and materials

### 2.1 Materials and solutions

The steel used herein was AISI 904L austenitic stainless steel with the following chemical composition (wt%): C = 0.016, Cr = 19.68, Ni = 24.08, Mo = 4.293, Mn = 1.470, Si = 0.292, S < 0.15, P = 0.027, N = 0.084, Cu = 1.273, and Fe balance. Samples (*Φ* 15 mm × 6 mm) were cut from a hot rolling plate, ground, mirror polished, and cleaned with acetone before testing.

The test solutions had different molar concentrations of HF (0.1, 1, 5, and 10 M) and HCl (0.1 and 1 M). The temperature was 50 °C for all the tests.

### 2.2 Electrochemical tests

A classical three-electrode cell, with a saturated calomel electrode (SCE) as the reference electrode and platinum foil as the counter electrode, was used for the electrochemical tests. For achieving steady-state condition, the samples were immersed in the solution for 30 min before the open circuit potential tended toward stability. The test results were obtained five times to ensure accuracy.

#### 2.2.1 Polarization curves and electrochemical impedance spectroscopy

The scanning rate of the polarization curve was 0.5 mV s^−1^ at a scanning range from −1.5 to +1.5 V, with a relative open circuit potential. Electrochemical impedance spectroscopy (EIS) was performed in the frequency range between 10^5^ and 10^−2^ Hz with voltage amplitude ±10 mV.

#### 2.2.2 Mott–Schottky test

The measurement frequency of the Mott–Schottky curve was 1000 Hz and the AC amplitude was 5 mV. The scanning range was from −0.2 V to 0.6 V for the relative reference electrode in HF, the potential increment being 0.05 V.

Based on the Mott–Schottky theory,^9^ the semiconducting characteristics of passive films can be explored by measuring the capacitance of the interface developed between the passive film and the Helmholtz layer, as a function of the applied electrode potential, and is given by the following equation:1
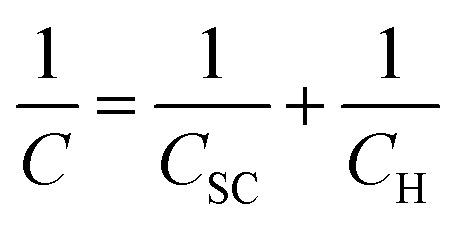
where *C*_SC_ and *C*_H_ represent the space charge and the Helmholtz capacitance, respectively.

However, since the capacitance of the space charge layer is much lower than that of the Helmholtz layer, the measured interfacial capacitance can be regarded as that of the space charge layer when the potential perturbations are applied with sufficiently high frequency.^10^ According to this theory, Morrison^11^ obtained the following relationships between the total capacitance and the potential difference for passive films on metal. Thus, the space charge capacitances of n-type and p-type semiconductors are given by eqn (2) and (3), respectively:2
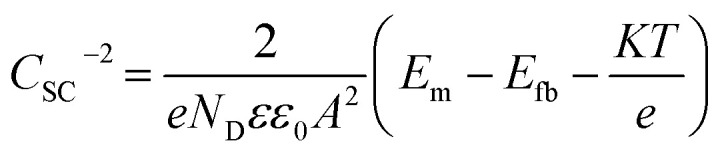
3
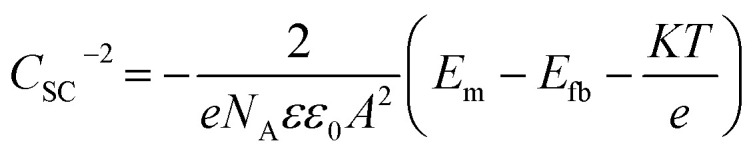
where *E*_m_ is the applied potential, *E*_fb_ is the flat band potential, *N*_D_ and *N*_A_ are the acceptor and donor density in the passive film, respectively, *ε*_0_ is the vacuum dielectric constant (8.85 × 10^−14^ F cm^−1^), *A* is the effective electrode area, *K* is the Boltzmann constant (1.38 × 10^−23^ J K^−1^), *e* is the charge (1.6 × 10^−19^ C), and *ε* is the relative permittivity. The general value corresponds to the composition of the passive film; for the sake of discussion, *ε* was taken as 15.6 for non-quantitative representation.^12^

Therefore, the validity of the Mott–Schottky analysis is based on the assumption that the capacitance^13^ of the space-charge layer is much lower than that of the Helmholtz layer and the data points on the *C*_SC_^−2^*vs. E* plot can describe the semiconducting behavior of the depletion region. *N*_D_ and *N*_A_ can be determined from the slope of this plot.

### 2.3 Phase analysis

When the sample was corroded in HF, a layer is loosely deposited on the surface of the sample. In the wet state, the deposited layer was scraped off with a glass slide, dried in a vacuum oven, and the powder on the slide was collected in a bottle. Certain amounts of powder and ethanol were placed in a beaker and were subjected to ultrasonic shock for 10–30 min. A capillary glass tube was used to absorb evenly mixed droplets, which were then applied on a copper wire. After the ethanol was volatilized, the sample was subjected to transmission electron microscopy (TEM) (Tecnai G2 F20 S-TWIN field-emission TEM). In addition, a part of the powder was used for X-ray diffraction analysis.

## 3. Results

### 3.1 The passivation behavior of 904L in HF

#### 3.1.1 Polarization curves

**Fig. 1:**
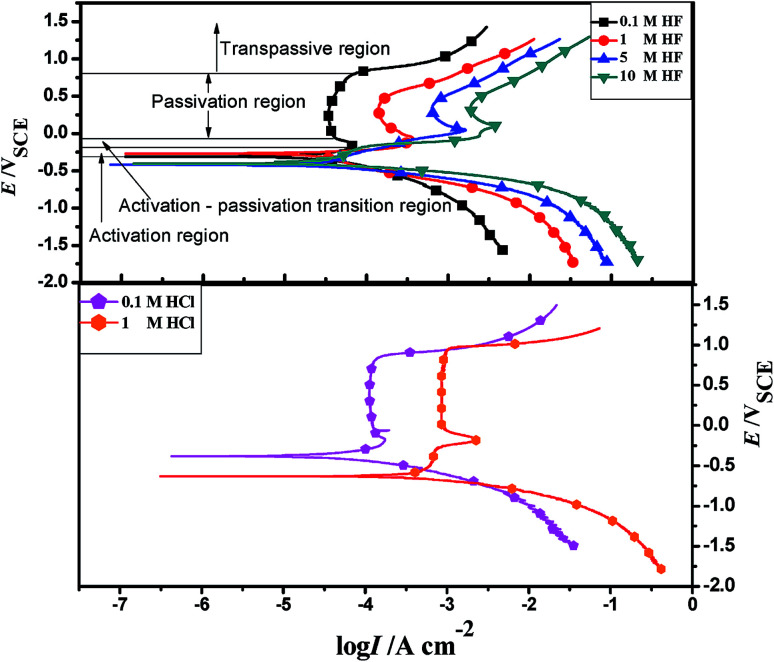
The polarization curves of 904L in HF and HCl solution.

Fig. 1 shows the polarization curves of 904L stainless steel in HF and HCl solutions of different concentrations at 50 °C. The corresponding polarization parameters determined from the extrapolation of polarization curves are listed in Table 1. Although the polarization curves were similar in HF, the curves obtained from the samples showed slight change with increase in concentration. The hydrogen evolution reaction occurred in the cathode region below the corrosion potential. With increasing HF concentration, the initial current density of the cathode polarization curve increases. Fig. 1 shows that the anodic polarization process is divided into the following four zones at 0.1 M. (i) Activation region: from the corrosion potential of −0.31 V to the critical passivation potential of −0.17 V; current density gradually increased in the process. (ii) Activation–passivation transition region: between −0.13 V and −0.04 V; the current density gradually decreases in this region and began to form a passivation film. (iii) Passivation region: −0.04 V to 0.855 V; the current density remained basically unchanged in this region, and the dimensional current density is less than the critical current density. (iv) Transpassive region: above 0.855 V; the passivation film is punctured and the current density increases in this region. Polarization curves of other concentrations have similar characteristics, and the anodic polarization regions are composed of four regions.

**Table 1:** Polarization parameters of 904L in HF and HCl solution

	*E*_corr_ (mV)	*I*_corr_ (μA cm^−2^)	*E*_b_ (V)	Corrosion rate (mm A^−1^)
0.1 M HF	−309.97	15.715	0.855	0.18484
1 M HF	−282.87	20.631	0.614	0.24267
5 M HF	−423.01	25.334	0.572	0.29798
10 M HF	−404.56	27.856	0.561	0.32765
1.1 M HCl	−380.54	54.496	0.835	0.64099
1 M HCl	−639.91	751.721	0.944	8.84181

Table 1 shows that the self-corrosion current density of 904L stainless steel in 0.1 M HF is only 15.715 μA cm^−2^. The self-corrosion current density and the passivation current density of 904L increase with increasing HF concentration. In addition, the passivation zone is gradually reduced as the concentration increases.

The 904L anodic polarization curves in HCl and HF are the same; they also have four regions. However, the passivation current density in HCl remained almost constant in the passive potential range, while that in HF changed constantly. This proved that the 904L passivation film in HCl is more stable than that in HF. Cid *et al.*^14^ found that nickel in HF was adsorbed preferentially on the metal surface with fluoride ions. Given that the nickel content in 904L is second only to iron, the deposited layer is likely to be nickel fluoride, which may hinder oxide film formation. The initial observation of the sample revealed that the sample surface would indeed have a layer of black precipitate after corrosion by HF, which was easily peelable, and the black deposits would increase with increasing concentration.

#### 3.1.2 Electrochemical impedance analysis

**Fig. 2:**
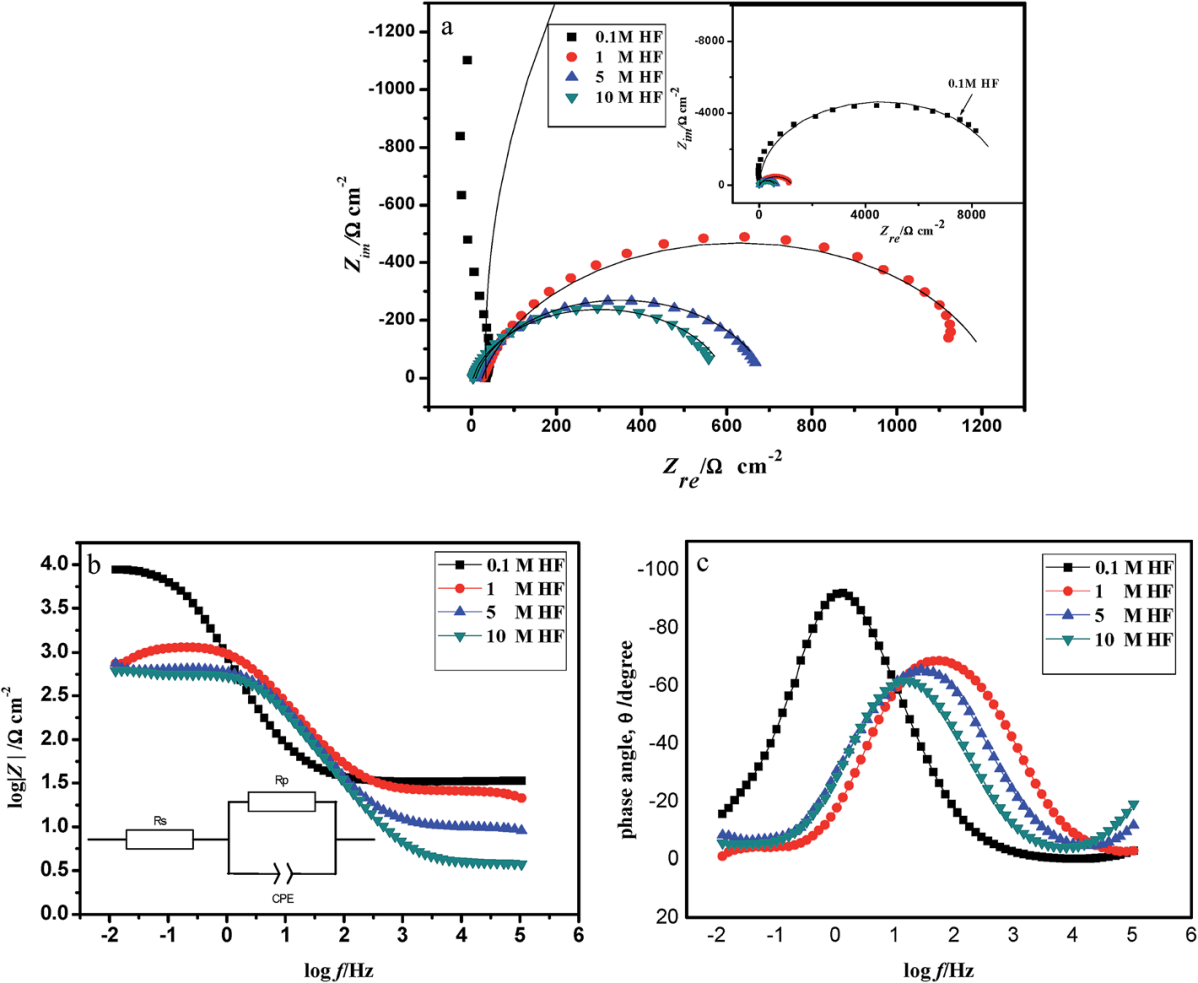
EIS diagrams of 904L in HCl and HF solution. (a) Nyquist representation; (b) and (c) Bode representation.

Fig. 2a shows the open-loop potential stabilities in the Nyquist plots of 904L stainless steel measured at different concentrations of HF. Fig. 2b and c show the Bode plots for 904L stainless steel at different concentrations of HF. The Nyquist plots showed depressed capacitive arcs for all the studied materials, suggesting analogous electrochemical behavior for all the samples. The size of the capacitance arcs reflects the charge transfer resistance during the corrosion process. The greater the radius of the capacitive loop, the greater is the charge transfer resistance. The Bode diagram (Fig. 2b) shows that the phase angle corresponds to the solution resistance between the working electrode and the reference electrode when the high-frequency region is close to 0°. In addition, only one peak appears in the phase angle and frequency diagram, and appears as a time constant. The impedance data were fitted using the equivalent circuit shown in Fig. 2b,^15–17^ where *R*_p_ represents the polarization impedance, *R*_s_ represents the solution resistance, and the constant-phase element is represented by the CPE, which is used to account for the capacitive response in the non-ideal state of the interface.

The values obtained after fitting (Table 2) show that the polarization resistance value is maximum in 0.1 M HF and *R*_p_ decreases 0.064 times when the HF concentration increases from 0.1 M to 10 M.

**Table 2:** The results of EIS of 904L in HF solution

	*R*_S_/Ω cm^−2^	*R*_P_/Ω cm^−2^	CPE/μF cm^−2^	*n*
0.1 M HF	35.19	9082	106.63	0.97
1 M HF	25.69	1208	112.15	0.84
5 M HF	10.24	668	116.49	0.86
10 M HF	4.01	584	124.65	0.86

In general, the capacitance represents the density of the passivation film. Table 2 shows that the capacitance gradually increases with increasing HF concentration, *i.e.* the passivation film becomes increasingly loose. This phenomenon occurred because fluoride ions preferentially form fluoride on the stainless steel surface in HF, and the fluoride adhering to the sample surface destroys the original oxide film on the sample surface while preventing the film from maintaining its activity. In addition, the protective properties of the electrode surface film are degraded. With increasing concentration, the surface-active points and the fluoride content on the electrode surface both increase. Consequently, the electrode surface is enriched with fluorine; the infiltration of fluoride ions leads to an increase in the conductivity of the passivation film and decrease in the density of the film, thereby accelerating the chemical dissolution of the film surface, resulting in fewer regions of the passivation film. Therefore, the measured resistance decreases, whereas the interface capacitance increases with increasing concentration.

### 3.2 Semiconducting properties of the passive film

**Fig. 3:**
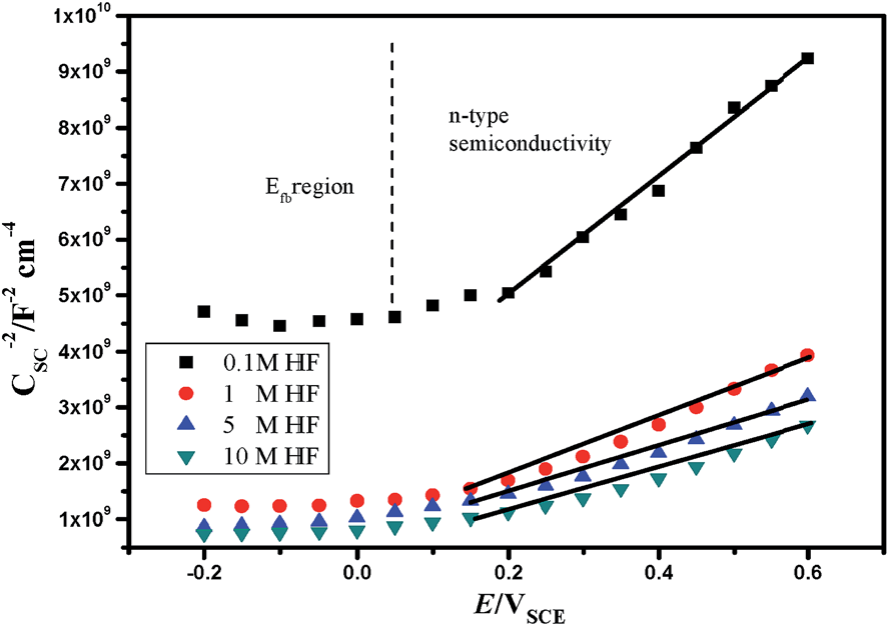
The Mott–Schottky curves of 904L in hydrofluoric acid.

The Mott–Schottky curve of 904L stainless steel at different concentrations of HF (Fig. 3) shows that all curves contain a straight line with a positive slope, characteristic of an n-type semiconductor. According to MacDonald's point defect model,^6^ the entire passivated film structure is dominated by anion vacancies belonging to n-type semiconductors.

**Fig. 4:**
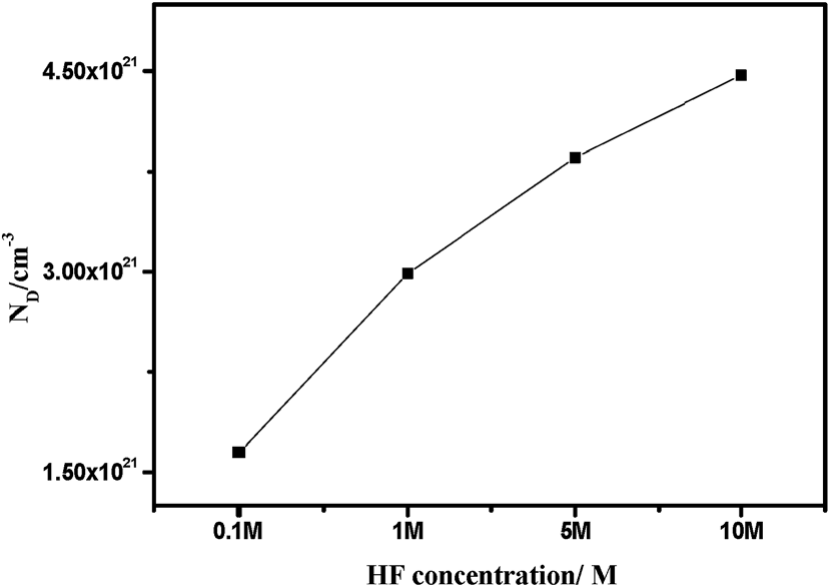
The density calculation of magazine donor of the passive film of 904L stainless steel.

The relationship between *N*_D_ and the slope of the *C*_SC_^−2^*vs. E* pattern can be obtained using eqn (4):4
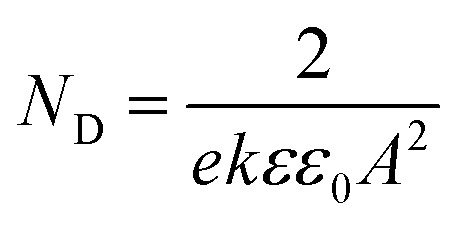
where *k* is the slope and the results are shown in Fig. 4. Fig. 4 shows that carrier concentration increases with increasing HF concentration, indicating that the impurity defect in the passivation film increases. Analysis of the binding impedance spectrum and the polarization curve shows that the increase in impurity defect is due to the accelerated accumulation of deposits with increasing HF concentration, leading to a reduction in the passivation film formation area.

### 3.3 Morphology analysis

**Fig. 5:**
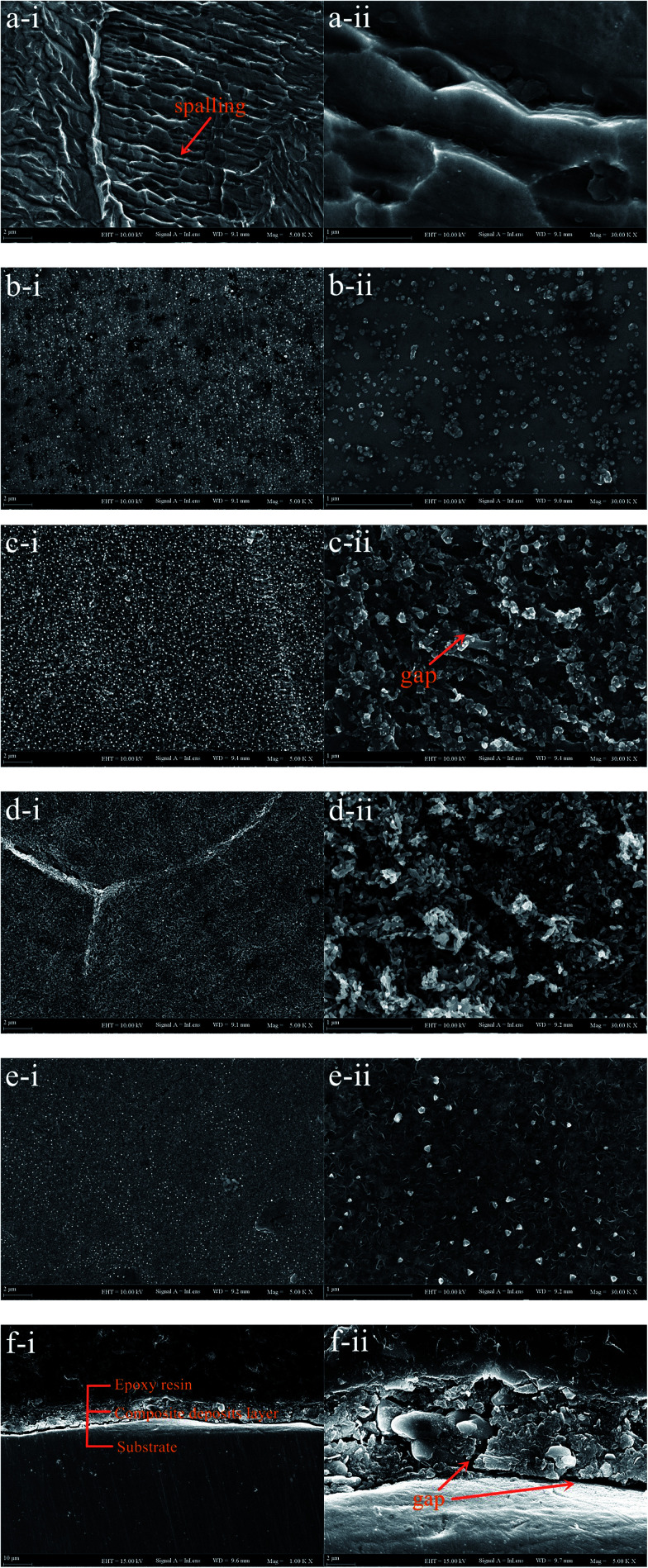
Corrosion morphology of 904L surface after immersion in 1 mol HCl (a),0.1 mol HF (b), 1 mol HF (c), 5 mol HF (d), 10 mol HF (e) and cross-section graph of the deposits after immersion in HF (f).

904L austenitic stainless steel was immersed in 100 mL of 1 M HCl solution and HF solutions of different concentrations (0.1, 1, 5, and 10 M) at 50 °C for 100 h. Fig. 5 shows the SEM images of the 904L surface after immersion in 1 M HCl solution and HF solutions of different concentrations at different magnifications. The surface of the corrosion sample after immersion in HCl shows obvious spalling phenomenon, and the surface appears smooth without any deposited layer (Fig. 5a). No corrosion can be observed on the surface of the corrosion sample after immersion in HF (Fig. 5b–e) and only the deposited layer is found (Fig. 5f).

The morphological analysis supports the deposited layer formation on the sample surface after immersion in HF. In addition, the inside of the deposited layer has an approximately net-like structure, the deposited layer is not dense, and there are many gaps inside the deposited layer. Simultaneously, many gaps exist between the bottom of the deposited layer and the substrate (Fig. 5c-ii and f). With increasing HF concentration, the amount of corrosion products on the sample surface increases and the deposited layer becomes denser. When the concentration increases to 10 M, the delicate structures of the deposits are not observed (Fig. 5b–e).

### 3.4 Phase analysis

To investigate the composition of the deposited layer, the deposits on the surface of the corrosion sample are collected for TEM and X-ray diffraction (XRD) studies. The collection method has been described in detail above to ensure that the collected deposits do not contain a matrix.

**Fig. 6:**
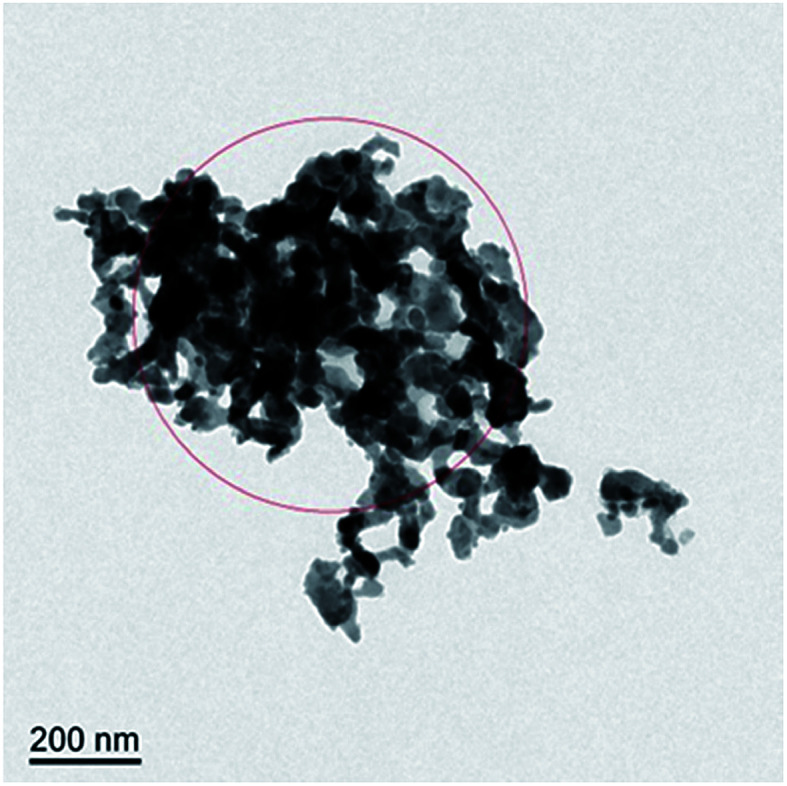
Transmission electron microscope of the deposits on the 904L surface after immersion.

**Fig. 7:**
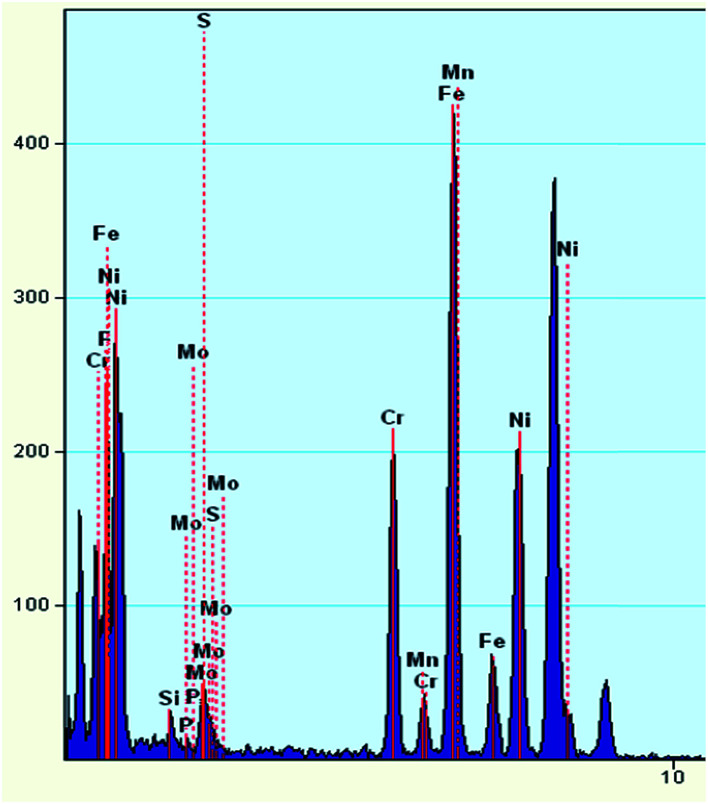
EDS spectrum of deposits on the 904L surface after immersion.

**Fig. 8:**
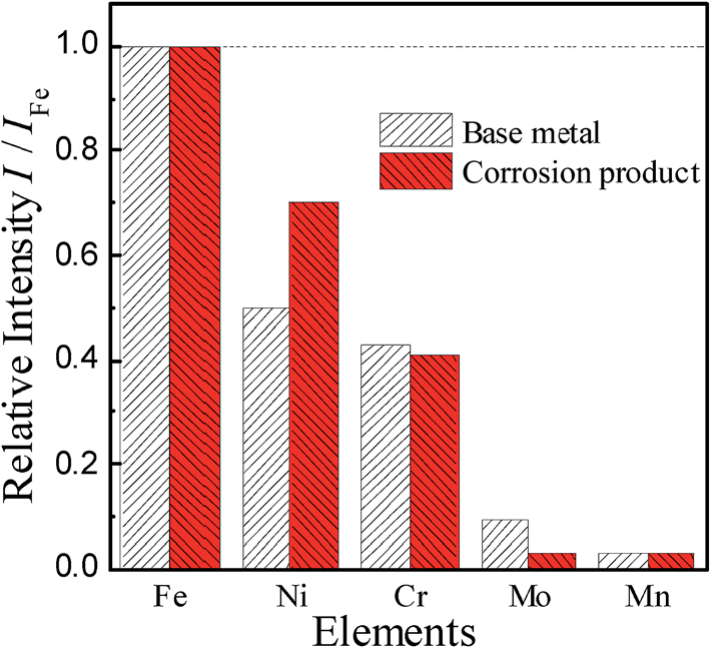
Elemental composition of the sediments of 904L in HF solution is compared with the original sample.

A TEM image of the deposit on 904L (Fig. 6) shows that each area is composed of many powdered deposits. Fig. 7 shows that iron, chromium, and nickel are the main elements of the deposits. The plot of the elemental composition of the deposit *versus* the elemental composition of the matrix is shown in Fig. 8, where all the elements are normalized with respect to elemental iron.

The difference between the ratio of the Cr and Mo contents in the matrix and the deposits are very small and even negligible. The ratio of Mn content did not change, and only the Ni content ratio increased significantly from 0.499 to 0.701, indicating that Ni enrichment in the deposits. Thus, nickel compounds account for the major part of the deposits; however, the Cr and Fe contents cannot be ignored.

**Fig. 9:**
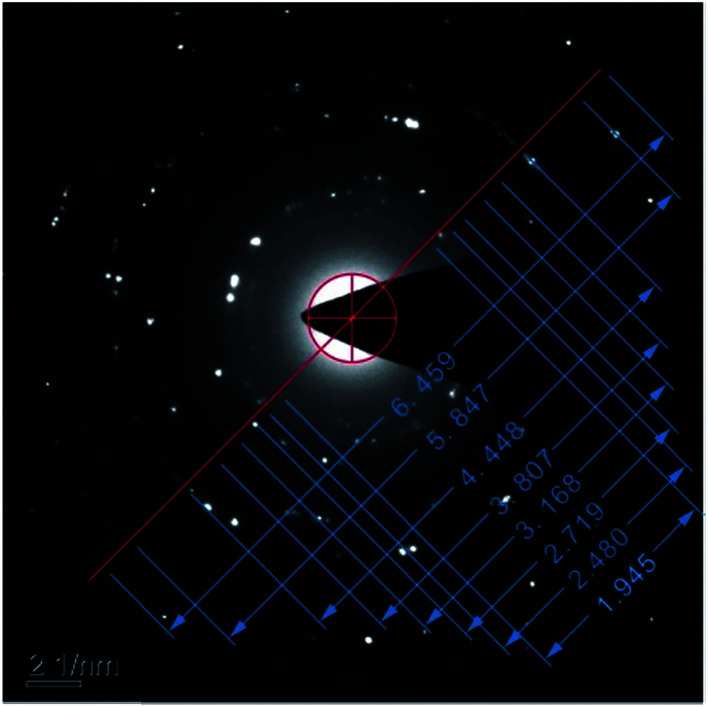
Electron diffraction pattern of the deposits.

**Fig. 10:**
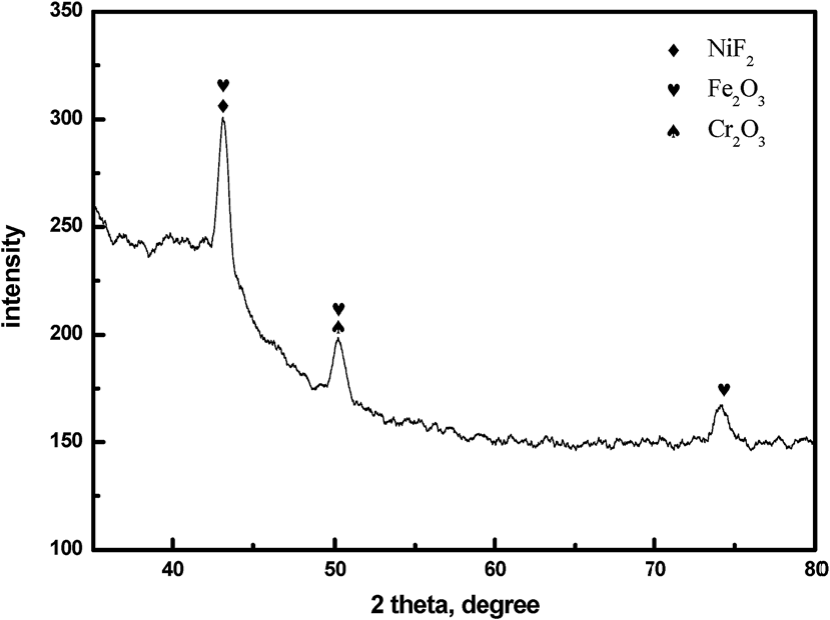
XRD patterns of the deposits formed on 904L.

**Fig. 11:**
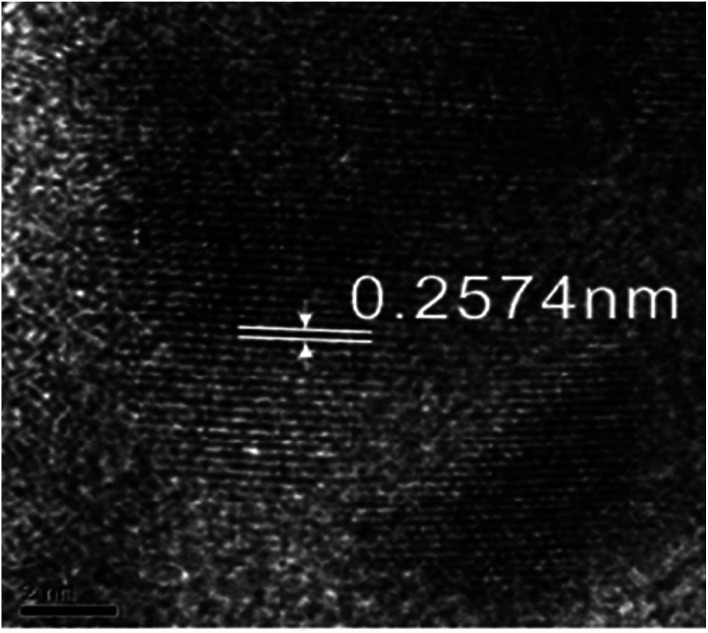
HREM micrograph of the deposits.

Fig. 9 shows the electron diffraction patterns of the collected deposits. Calibration of the electron diffraction spots shows that the radius ratio of the diffraction ring is not consistent with the extinction law of any crystal system; therefore, the corresponding compound can only be found by the *d* value. The EDS pattern (Fig. 7) is used to determine the possible compounds for the element. The obtained results (Table 3) showed that the deposits are NiF_2_, Fe_2_O_3_, and Cr_2_O_3_. This result is consistent with the XRD analysis (Fig. 10). Fig. 11 shows a high-resolution image of the deposits, where each point represents an atom, and the distance between each atom represents plane spacing. The calculated interplanar spacing is approximately the same as that of NiF_2_ (0.2574 nm). This result further confirms that the deposits are predominantly composed of nickel fluoride.

**Table 3:** Experimental and calculated *d*-spacing values for the diffraction rings of Fig. 9

Diffraction ring sequence number	*d*_test_/Å	*d*_standard_/Å	*hkl*	Compound
1	2.5707	2.570	101	NiF_2_
2	2.2016	2.201	113	Fe_2_O_3_
3	1.8389	1.838	024	Fe_2_O_3_
4	1.5782	1.579	122	Cr_2_O_3_
5	1.3134	1.310	119	Fe_2_O_3_
6	1.1241	1.123	134	Cr_2_O_3_
7	0.8551	0.856	303	NiF_2_
8	0.7741	0.774	600	NiF_2_

## 4. Discussion

Fig. 1 shows that the polarization curve of 904L stainless steel in HF has a passivation region that is not as stable as that in hydrochloric acid, and the passivation current changes constantly. This shows that although a passivation film can be produced on the 904L surface, the formation is hindered due to the deposits on the 904L surface in HF (Fig. 5). In addition, the inside of the deposited layer has an approximately net-like structure, the deposited layer is not dense, and there are many gaps inside the deposited layer. Moreover, many gaps exist between the bottom of the deposited layer and the substrate (Fig. 5c-ii and f). These gaps provide the conditions suitable for producing a passive film on the 904L surface. Fig. 8 shows that iron, chromium, and nickel are the main elements in the deposits. The deposited layer is composed of NiF_2_, Fe_2_O_3_, and Cr_2_O_3_, determined by the calibration of the electron diffraction spot diagram (Fig. 9). Since the iron and chromium oxides are both components of the passivation film, both these oxides can be produced in both HF and HCl on the surface of 904L. Thus, the deposition of nickel fluoride is a major factor impeding the production of passive film.

**Fig. 12:**
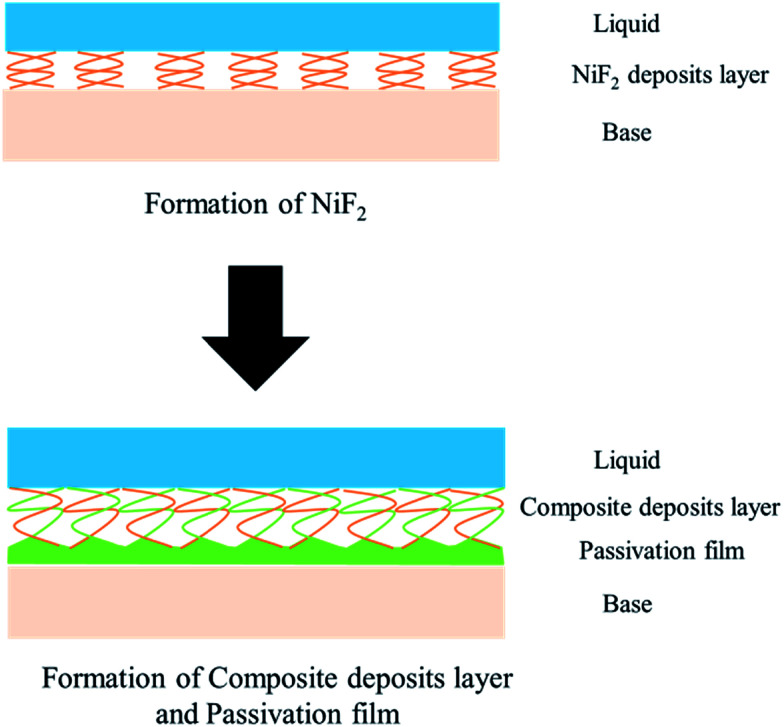
The evolution of the double-layer structure on 904L surface in HF solution.

From what has been discussed above, the evolution model (Fig. 12) can be established. When 904L stainless steel was immersed in HF, the original oxide film of the surface was destroyed, the sample surface began to dissolve, and fluoride ions of the solution react with nickel on the metal surface to produce nickel fluoride precipitates. With the reaction of nickel and fluoride ions, iron and chromium were precipitated one after another. Since the fluoride ions have been depleted, iron and chromium can only react with the oxygen ions in the solution to form oxides. Due to the deposition of nickel fluoride on the 904L surface, large amounts of iron oxide and chromium oxide are mixed with nickel fluoride to form a mixed deposition layer. A very small portion of the iron oxide and chromium oxide occurs at the bottom of the deposition layer through the deposition gap to produce a dense passive film that covers the surface of 904L stainless steel. The deposited layer becomes denser with increasing HF concentration due to the accelerated accumulation of deposits, leading to a reduction in the passivation film formation area. The deposited layer is formed by the accumulation of solid products; therefore, its density is not as high as that of the passivation film, and corrosion resistance of 904L decreases with increasing HF concentration.

## 5. Conclusions

(i) An insoluble layer is deposited on 904L in HF due to a preferential reaction between [F^−^] and [Ni] from the alloy. The insoluble deposited layer helps isolate the aggressive ions from the base metal, and inhibits passivation of 904L in HF, the mechanism of which was entirely different from that in HCl.

(ii) The density of the deposited layer is not as high as that of the passivation film, and the corrosion resistance of 904L decreases with increasing HF concentration.

## Conflicts of interest

There are no conflicts to declare.

## Supplementary Material
